# Sensitivity of Different Clinical Outcome Measures in Assessing Adults With Becker Muscular Dystrophy: A 3-Year Natural History Study

**DOI:** 10.1212/WNL.0000000000214071

**Published:** 2025-09-05

**Authors:** Esther J. Schrama, Zaïda Koeks, Nienke M. Van De Velde, Iris Alleman, Jules J. van Benthem, Pieteke W. van Weperen, Melissa T. Hooijmans, Hermien E. Kan, Pietro Spitali, Nina Ajmone Marsan, Douwe E. Atsma, Hermine A. van Duyvenvoorde, Jan J.G.M. Verschuuren, Erik H. Niks

**Affiliations:** 1 Department of Neurology, Leiden University Medical Center, the Netherlands;; 2 Department of Physiotherapy, Reinier de Graaf Ziekenhuis, Delft, the Netherlands;; 3 Department of Rehabilitation, University Medical Center Utrecht, the Netherlands;; 4 Department of Orthopaedics, Rehabilitation and Physiotherapy, Leiden University Medical Center, the Netherlands;; 5 Amsterdam Movement Sciences, Sports, the Netherlands;; 6 Department of Radiology and Nuclear Medicine, Amsterdam University Medical Centers, University of Amsterdam, the Netherlands;; 7 Department of Radiology, C.J. Gorter MRI Center, Leiden University Medical Center, Netherlands;; 8 Duchenne Center Netherlands;; 9 Human Genetics Department, Leiden University Medical Center, the Netherlands;; 10 Department of Cardiology, Heart Lung Center, Leiden University Medical Center, the Netherlands; and; 11 Department of Clinical Genetics, Leiden University Medical Center, the Netherlands.

## Abstract

**Background and Objectives:**

Slow and highly variable disease progression in Becker muscular dystrophy (BMD) stresses the need to develop sensitive outcome measures for clinical trials. We evaluated responsiveness of different outcome measures in adult patients with BMD over 3 years and explored if the sensitivity of outcome measures can be increased by selecting on phenotype or genotype.

**Methods:**

Genetically confirmed patients with BMD were recruited via the Dutch Dystrophinopathy Database. Functional tests included North Star Ambulatory Assessment (NSAA), Timed Tests, Performance of the Upper Limb version 1.2, and pulmonary function yearly and echocardiography biennially. Mean changes and standardized response means (SRMs) were calculated per year. Outcome measures with SRM ≥0.80 were considered to have a high responsiveness to change. Two genetic subgroups—deletion of exons 45–47 and variants affecting the neuronal nitric oxide synthesis (nNOS) binding site—and one functional subgroup—NSAA between 10 and 32 at baseline—were analyzed separately.

**Results:**

Thirty-six patients with BMD were included (mean age 41.2 years, range 18.6–67.3, 27 ambulant at baseline). A high responsiveness was observed for the rise from floor velocity (RFFv) at 3-year follow-up (SRM −0.91, n = 16), while the SRMs for the other outcome measures were <0.8 at all time points. In the functional subgroup, a high responsiveness was observed for RFFv at 1-year follow-up (SRM −0.82, n = 10), along with 4-stair climb velocity (4SCv) (SRM −1.03, n = 11) and NSAA (SRM −0.82, n = 13) at 2-year follow-up. Genetic subgroups did not significantly differ in age at loss of ambulation (LoA). Upper limb and pulmonary function were preserved beyond LoA. Decline of cardiac function was independent of skeletal muscle function.

**Discussion:**

RFFv was the only outcome measure sensitive to change at 3-year follow-up. Selecting on phenotype resulted in a high responsiveness for RFFv at 1-year follow-up and for 4SCv and NSAA at 2-year follow-up. NSAA could be performed in more participants compared with RFFv and therefore seems preferable in clinical trials for ambulant patients. Despite limitations in sample size, the results suggest that clinical trials in the ambulant population could be enriched by selecting on phenotype.

## Introduction

Becker muscular dystrophy (BMD) is an X-linked recessive disorder caused by pathogenic variants in the dystrophin (*DMD*) gene leading to reduced levels of truncated dystrophin.^1^ Dysfunction of the dystrophin protein increases susceptibility of muscle fibers to contraction-induced damage resulting in fibrosis and replacement of muscle tissue by fat. The prevalence of BMD is estimated to be around 1.6 per 100.000 people.^2^ The severity of muscle weakness in patients with BMD is highly variable, and clinical symptoms range from severe muscle weakness in childhood, overlapping with Duchenne muscular dystrophy (DMD), to only mild weakness or muscle cramps in the elderly.^3-6^ Cardiac involvement, predominantly cardiomyopathy, occurs in about 70% of the patients with BMD.^7-9^ Respiratory function generally declines slowly, with a more rapid decline after loss of ambulation (LoA).^10^

Causes for disease variability in BMD are not fully understood. The quantity of remaining dystrophin protein produced has been studied as a possible explanatory factor for the clinical severity. Although thresholds have been suggested in which dystrophin levels below 10% of normal were associated with a more severe phenotype and above 40% with a mild phenotype, no evident correlation between dystrophin quantity and disease severity could be established.^11-14^ In addition, it has been shown that dystrophin expression in patients with BMD is inhibited by inflammation-induced microRNAs, suggesting a negative feedback loop induced by chronic inflammation resulting in disease progression.^15^ Several genotype-phenotype relations have been proposed; a relatively mild phenotype was described in patients with a deletion ending at exon 51 or with a single exon 48 deletion, while a deletion starting at exon 45 has been reported to result in a more severe phenotype.^16,17^ In addition, the region of the dystrophin protein that is impaired (e.g., central rod domain, hinge region) has been associated with the age of onset of cardiomyopathy.^18^ Finally, pathogenic dystrophin variants affecting the neuronal nitric oxide synthase (nNOS) binding site (encoded by exon 42 to 46) have been proposed to result in a more severe phenotype.^19^ Binding of nNOS to dystrophin and synthesis of nitric oxide (NO) is crucial for regulating the delivery of local oxygen and nutrients by inducing vasodilatation as well as angiogenesis.^20^ Studies with biopsies of patients with DMD and nNOS-deficient mice also suggest dysfunction of NO contributes to muscle injury in dystrophinopathies.^21^

The low prevalence and high disease variability presents a paramount complication in the design of clinical trials. Slow deterioration limits the chance of observing clinically relevant changes within the duration of a clinical trial, which typically lasts between 1 and 2 years. There is thus an urgent need to identify clinical outcome measures reflecting disease progression and showing a relation to the clinically significant end points beyond the duration of a trial. Longitudinal data on natural history in BMD are sparse, and detailed information about additional factors influencing the variability of phenotype and rate of disease progression is needed. In this study, we describe a cohort of 36 patients with BMD followed for 3 years (baseline and 3 follow-up visits). We aimed to identify outcome measures responsive to change and explore if that responsiveness can be increased by selecting groups based on phenotype or genotype.

## Methods

### Standard Protocol Approvals, Registrations, and Patient Consents

The study was approved by the medical ethical committee of the Leiden University Medical Center (LUMC), the Netherlands (P14.243). Written informed consent was obtained before enrolment.

### Participants and Study Design

Participants were recruited between October 2014 and June 2016 through the Dutch Dystrophinopathy Database (DDD).^22^ The DDD is the national registry aimed to include all dystrophinopathy patients in the Netherlands, independent of their center for clinical care. By registering, participants consent to receive information on clinical studies actively recruiting. All adult patients registered in the DDD with the diagnosis BMD were informed without selection. Male patients with BMD aged 18 years and older were included, either with an in-frame variant in the *DMD* gene or with an out-of-frame variant known to be associated with BMD and with preserved ambulation beyond 16 years of age without steroid treatment. Genetic testing reports of all participants were reviewed by a clinical laboratory geneticist of the LUMC. Participation in an interventional trial was the sole exclusion criterium.

### Assessments

Participants were invited for 4 yearly study visits. A complete medical history of each participant was obtained and updated during each study visit, including age at first symptoms, type of first symptoms, age at diagnosis, use of medication, comorbidities, use of ventilatory support, the occurrence of functional disabilities, and the use of a walking aid or wheelchair. LoA was defined as the inability to walk 10 m with support of a cane. Weight and height were recorded at each visit, and body mass index (BMI) was calculated using these measures.

Functional tests included the North Star Ambulatory Assessment (NSAA), Performance of Upper Limb (PUL) version 1.2, and timed tests: 6-minute walk test (6MWT), 10 m walk/run test (10MWRT), rise from floor (RFF) and 4-stair climb (4SC), and 4-stair descend (4SD). Velocities were calculated for the timed tests to allow analysis of the decline and loss of function. Velocities of zero were entered when a participant was unable to perform a given test. All functional tests were performed by trained evaluators.

We monitored pulmonary function by measuring Forced Vital Capacity % predicted (FVC%pred) using a handheld spirometer (Microloop, CareFusion). Cardiac function was assessed by measuring left ventricular ejection fraction (LVEF) and left ventricular global longitudinal strain (LV GLS) twice at a 2 year interval using echocardiography by an experienced imaging cardiologist at the cardiology department of the LUMC. We strived to perform the first echocardiography at baseline. However, when a echocardiography was performed as part of the standard clinical care for LUMC patients participating in this study, the echocardiography was not repeated at study visits to minimize the burden for participants. Therefore, the echocardiography did not always exactly coincide with the first and the third visit. Cardiac function was considered to be impaired at baseline when the LVEF within 6 months of the first visit was <52%. In case no echocardiography was available at baseline, cardiac function was considered impaired if participant was using medication prescribed for cardiomyopathy.

We performed 2 subgroup analyses based on genotype and 1 on phenotype. The first subgroup analysis consisted of participants with the most common deletion of exons 45 to 47 (del 45–47 vs others). The second included participants who had variants encompassing at least 1 exon in the region encoding for the nNOS binding site at exons 42 to 46 in the *DMD* gene (nNOS− vs other). In the phenotype subgroup analysis, participants were grouped based on the NSAA score obtained at first visit: lower than 10, 10 to 32 inclusive, and 33 or 34.

### Statistical Analysis

Statistical analysis was performed using SPSS version 29.0 (IBM, Armonk, NY). Analyses were conducted using available data; missing data were not imputed or otherwise adjusted. For comparison of subgroups at baseline, *t* tests and χ^2^ tests were used. The standardized response means (SRMs) were used to assess responsiveness of outcome measures over time, and were calculated as the mean change over follow-up divided by the SD of the change.^22^ When a participant was unable to perform a timed test at all visits, he was excluded from the SRM analysis. If the participant was able to perform the test at baseline, but lost the ability to perform at a follow-up visit, the first visit unable to perform was included as a zero and any further visits were excluded from the analysis. An outcome measure with SRM ≥0.80 was considered to have a high responsiveness.^23^ Subsequently, Lehr formula was used to calculate the required sample size (SS) of an interventional trial to demonstrate an effect of treatment based on the SRM.^24^ We assumed the hypothetical treatment would lead to a 50% reduction in disease progression in a 24 month trial with a power of 80%, and an α < 0.05 in a 1:1 randomization. Survival curves of age at first symptoms, diagnosis, and LoA for the different subgroups were tested for equality of survival distribution using a log-rank test. The association between decline in functional tests with decline in cardiac function was assessed using a linear mixed effects model in RStudio version 2024.04.2. The participant ID was included as a random effect. The LV GLS and NSAA or RFFv were included as fixed effects. Data were included if echocardiography and functional tests were recorded up to 6 months apart. The results were considered significant at *p* < 0.05.

### Data Availability

Anonymized data can be made available to qualified investigators on request.

## Results

Ninety-two people were contact through the DDD, of whom 36 BMD patients from 33 unrelated families participated.^25^ All familial cases were brother pairs. Twenty-eight participants completed all 4 visits. The other participants completed a varying number of visits: the first visit only (2), the first 2 visits (3), the first 3 visits (1), the first and the fourth visit (1), or the first, third, and fourth visit (1). Reasons for missing study visits were illness (4), withdrawal of consent (2), participation in a medication trial (1), and deceased (1).

The age at first visit was between 18.9 and 67.3 years. Nine participants (25%) had lost ambulation prior to the first visit (range: 1 month to 29 years prior), 3 participants lost ambulation during follow-up. None of the participants had ever used corticosteroids. Twenty-three participants had cardiomyopathy at baseline, defined as LVEF <52% (18) or use or cardiac medication (5). Five participants had an implantable cardioverter-defibrillator and 1 a pacemaker. Descriptive statistics for the whole cohort at baseline are presented in Table 1.

**Table 1:** Characteristics of the Study Population

	All participants (N = 36)	Del 45–47 subgroup analysis		nNOS subgroup analysis		Functional subgroup analysis	
		Del 45–47 (n = 11)	Other (n = 25)	nNOS− (n = 21)	Other (n = 15)	NSAA 10–32 at baseline (n = 15)	Other (n = 21)
Age at first visit, y, mean (SD)	41.8 (12.8)	41.2 (11.4)	42.1 (13.7)	44.4 (12.1)	38.1 (13.3)	40.1 (14.6)	43.1 (11.7)
Age at first symptoms^a^, y, mean (SD)	9.0 (5.1)	8.5 (5.8)	9.3 (4.8)	8.8 (4.7)	9.4 (5.8)	8.4 (5.0)	9.5 (5.2)
Age at diagnosis, y, mean (SD)	20.1 (12.1)	20.2 (11.4)	20.1 (12.6)	21.0 (12.1)	19.0 (12.3)	20.7 (11.6)	19.8 (12.7)
Wheelchair use, n (%)							
Never	17 (47.2)	6 (54.5)	11 (44.0)	9 (42.9)	8 (53.3)	9 (60.0)	8 (38.1)
Intermittent	11 (30.6)	4 (36.4)	7 (28.0)	7 (33.3)	4 (26.7)	6 (40.0)	5 (23.8)
Permanent	8 (22.2)	1 (9.1)	7 (28.0)	5 (23.8)	3 (20.0)	0 (0.0)	8 (38.1)
Age at LoA^b^, y, mean (SD)	36.2 (13.7)	33	36.5 (14.3)	40.6 (12.7)	28.5 (13.2)	30	36.8 (14.2)
Cardiomyopathy, n (%)	23 (69.9)	7 (63.6)	16 (64)	13 (61.9)	10 (66.7)	12 (80)	11 (52.4)
Ventilatory support, n (%)	2 (5.6)	0 (0)	2 (8)	0 (0)	2 (13.3)	0 (0)	2 (9.5)
BMI at first visit, mean (SD)	25.1 (4.3)	23.2 (3.6)	25.9 (4.4)	24.6 (4.9)	25.7 (3.4)	24.3 (3.3)	25.7 (4.9)

Abbreviations: BMI = body mass index; LoA = loss of ambulation; nNOS = neuronal nitric oxide synthesis; NSAA = North Star Ambulatory Assessment.

a For 2 participants the age at first symptoms was unknown, neither of them had a deletion 45–47 or a variant involving exon 42–46, and both were included in the group other for the functional analysis.

b Eleven participants lost ambulation before or during the study period, 1 in the del 45–47 subgroup, 7 in the nNOS− subgroup, and 1 in the subgroup with NSAA 10–32 at baseline.

Pathogenic variants in the *DMD* gene included exonic deletions in 28 participants (78%), exonic duplications in 5 participants (14%), a nonsense variant in 2 participants (6%), and a missense variant in 1 participant (3%). In all duplications and in 26 deletions, the variants were predicted to be in-frame. Two participants carrying a variant predicted to be out-of-frame had a deletion of exon 3 to 7 and were ambulant beyond the age of 16: one was ambulant throughout the study and the other had lost ambulation at the age of 45.

Regarding the subgroups, 11 participants harbored a deletion of exons 45–47 (del 45–47), 21 participants harbored a variant likely to affect the nNOS binding site located at exons 42 to 46 (nNOS−), and 15 participants obtained a NSAA score between 10 and 32 at baseline. The variants likely to affect the nNOS binding site included 1 duplication of exon 14 to 42 and deletions of exons 30 to 44 (1), exons 45 to 47 (11), exons 45 to 48 (2), exons 45 to 49 (1), exons 45 to 53 (1), and exons 45 to 55 (4). A detailed description of genetic variants is presented in eTable 1. Baseline characteristics for the 3 subgroups are presented in Table 1. The subgroups did not differ in age, BMI, or the proportion with impaired cardiac function at baseline. Wheelchair use at baseline did not differ between the genetic subgroups.

**Figure 1:**
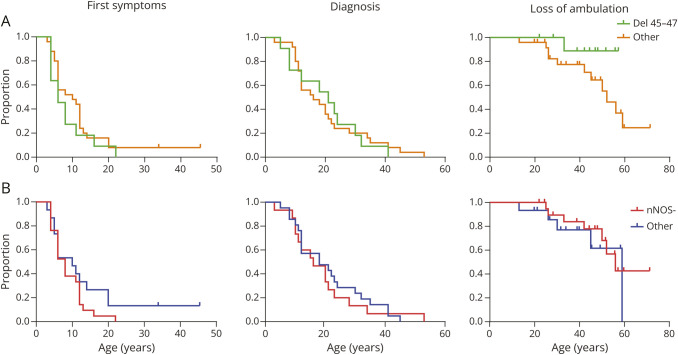
Kaplan-Meier Plots of Age at First Symptoms, Diagnosis, and Loss of Ambulation for the Genetic Subgroups Different colors indicate the groups of participants with a deletion of exons 45–47 (del 45–47) in green vs other pathogenic variants in yellow (A), those with a pathogenic variant predicted to affect the nNOS binding site (nNOS−) in red, and those with other pathogenic variants in blue (B). nNOS = neuronal nitric oxide synthesis.

To explore the genotype-phenotype relation in our cohort, we generated Kaplan-Meier plots of the age at first symptoms, age at diagnosis, and age at LoA grouped based on genetic variants (Figure 1). The log-rank tests were not significantly different for participants with a variant likely to affect the nNOS binding site (nNOS−, ρ = 0.208, ρ = 0.654, ρ = 0.541 for first symptoms, diagnosis, and LoA, respectively) or for participants with a deletion of exons 45 to 47 (del 45–47, ρ = 0.275, ρ = 0.922, ρ = 0.112, respectively). Twenty-two participants reported their first symptoms. The first symptoms that were reported most frequently were muscle cramps (8), muscle ache (6), toe walking (5), and difficulty walking (5) (eTable 2).

Functional tests were in general well tolerated and feasible; no major injuries occurred during any of the tests. On one occasion, the 10MWRT was not conducted because of a fall directly after the 6MWT, and on another occasion, the 6MWT was not conducted because of a near fall during the 10MWRT. In 2 participants, a note was added to not conduct the 10MWRT and 6MWT (1) or 4SC and 4SD (1) at the next follow-up visit because of a perceived risk of falling. Both proved then unable to perform the respective tests at the following visits due to muscle weakness. The 6MWT was ceased on 2 occasions, that is, the inability to complete the test (1) and pain in the hip region (1). Seven participants used a walking aid with the functional tests for stability. Two participants refused all ambulatory functional tests on 1 occasion (1) or all visits (1) because the burden of performing tests at maximum effort was considered too high. These 2 participants said they were anxious for muscle ache and fatigues in the days following the tests. One of them also refused the PUL on one occasion but completed the PUL during all follow-up visit and obtained maximum scores. Furthermore, one participant was unable to perform the PUL or any other functional test. He had limited muscle function remaining and was only capable of slight flexion and extension of the fingers and minimal movement in the feet and toes.

**Figure 2:**
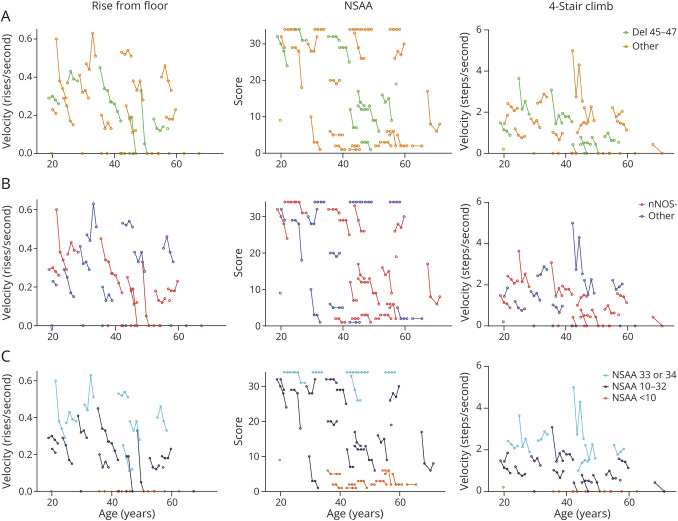
Individual Disease Trajectories for RFFv, NSAA, and 4SCv Different colors indicate the groups of participants with a deletion of exons 45–47 (del 45–47) in yellow vs other pathogenic variants in green (A), those with a pathogenic variant predicted to affect the nNOS binding site (nNOS−) in red, and those with other pathogenic variants in blue (B), and 3 groups based on baseline NSAA (C). A velocity of zero is assigned to participants who lost to ability to perform the test. 4SCv = 4-stair climb velocity; nNOS = neuronal nitric oxide synthesis; NSAA = North Star Ambulatory Assessment; RFFv = rise from floor velocity.

Individual trajectories on the RFFv, NSAA, and 4SCv are shown in Figure 2 and on the 4SDv, 10MWRv, and 6MWT in eFigure 1. Of the 7 participants with a NSAA score of 33 or 34 at first visit, 5 obtained unaltered maximum scores over 3 years of follow-up. A similar pattern was observed in the group with NSAA scores below 10, whereas in the middle group, the majority showed relatively consistent decline. In the RFF and 4SC, the participants with evident fluctuations in scores were mainly in de NSAA 33–34 subgroup. Visually, the most steady decline could be observed in the NSAA 10–32 subgroup, in contrast to any group based on genotype. Subsequently, SRMs and SSs were calculated for the whole cohort and for NSAA 10–32 subgroup (Table 2). SRMs were not calculated for subgroups based on genotype because no visually distinct decline was observed in these subgroups. None of the outcome measures showed a high responsiveness to change at 1 and 2 years of follow-up in the whole cohort. At 3 years of follow-up, a high responsiveness was observed for the RFFv (SRM −0.91, SS 77). Within the functional subgroup with NSAA scores of 10–32 at baseline, at 1-year follow-up, a high responsiveness was observed for the RRFv (SRM −0.82, SS 96) and at 2-year follow-up for RFFv (−0.94, SS 73), 4SCv (SRM −1.03, SS 60), and NSAA (SRM −0.82, SS 95). The high responsiveness observed for RFFv, 4SCv, and NSAA in this functional subgroup persisted at 3-year follow-up (SRMs −1.03, −1.10 and −0.86, respectively).

**Table 2:** Mean Performance at Baseline of the Ambulatory Tests and the Mean Change, SRM, and SS at 1-, 2-, and 3-Year Follow-Up

Functional test	Group	First visit		1-y follow-up				2-y follow-up				3-y follow-up			
		N	Mean (SD)	N	Mean change (SD)	SRM	SS	N	Mean change (SD)	SRM	SS	N	Mean change (SD)	SRM	SS
RFFv (rises/s)	All	18	0.33 (0.13)	17	−0.06 (0.09)	−0.65	153	16	−0.07 (0.11)	−0.58	189	16	−0.10 (0.11)	**−0.91**	77
	NSAA10-32	11	0.27 (0.10)	10	−0.07 (0.08)	**−0.82**	96	9	−0.09 (0.10)	**−0.94**	73	9	−0.11 (0.11)	**−1.03**	60
10MWRv (m/s)	All	25	1.97 (1.15)	22	−0.28 (0.41)	−0.68	138	20	−0.20 (0.41)	−0.48	273	20	−0.21 (0.52)	−0.39	414
	NSAA10-32	15	1.55 (0.68)	13	−0.21 (0.29)	−0.72	125	12	−0.14 (0.27)	−0.53	225	12	−0.12 (0.35)	−0.33	571
4SCv (stairs/s)	All	21	1.69 (1.18)	19	−0.40 (0.69)	−0.58	190	18	−0.32 (0.46)	−0.69	134	18	−0.54 (0.73)	−0.75	114
	NSAA10-32	13	1.26 (0.74)	12	−0.25 (0.50)	−0.50	255	11	−0.38 (0.37)	**−1.03**	60	11	−0.48 (0.44)	**−1.10**	53
4SDv (stairs/s)	All	21	2.24 (1.71)	19	−0.41 (1.13)	−0.36	486	18	−0.27 (1.83)	−0.15	3008	17	−0.40 (1.26)	−0.32	633
	NSAA10-32	13	1.55 (1.09)	12	−0.42 (0.70)	−0.60	177	11	−0.38 (0.74)	−0.51	244	10	−0.45 (0.60)	−0.74	117
6MWT (m)	All	25	423 (156)	24	−10 (72)	−0.14	3335	23	−22 (79)	−0.28	810	18	−19 (86)	−0.22	1322
	NSAA10-32	15	411 (121)	14	−30 (75)	−0.40	401	13	−37 (73)	−0.50	251	11	−53 (79)	−0.67	144
NSAA	All	32	18.94 (12.67)	28	−1.36 (2.45)	−0.55	209	26	−1.96 (2.86)	−0.69	136	26	−2.65 (3.87)	−0.69	136
	NSAA10-32	15	22.00 (8.27)	14	−2.14 (3.08)	−0.69	133	13	−2.77 (3.37)	**−0.82**	95	12	−4.25 (4.91)	**−0.86**	86

Abbreviations: 4SCv = 4-stair climb velocity; 4SDv = 4-stair descend velocity; 6MWT = 6-minute walk test; 10MWRTv = 10 m walk run velocity; NSAA = North Star Ambulatory Assessment; RFFv = rise from floor velocity; SRM = standardized response mean; SS = sample size.

SRMs >0.8 are in bold.

**Figure 3:**
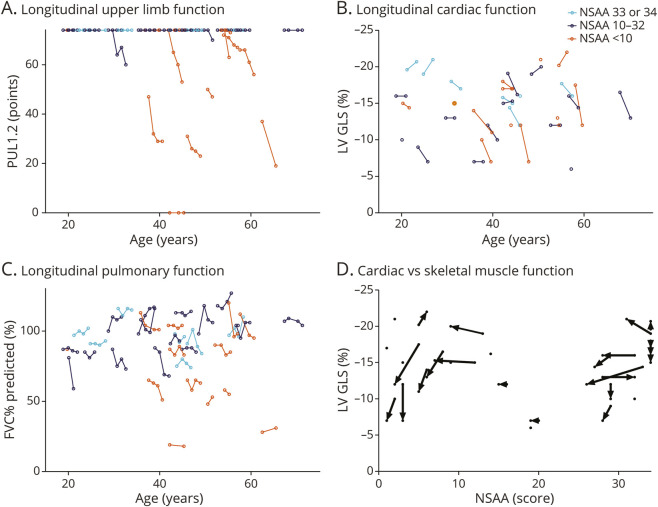
Longitudinal Cardiac, Pulmonary, and Upper Limb Function in BMD Patients Panels A–C show individual trajectories of the PUL1.2 (A), LV GLS (B), and FVC%pred (C), colored by NSAA score at first measurement. Panel D shows LV GLS quantified using ultrasound in relation to NSAA score at the corresponding time point. Arrows indicate the trajectory from first to second measurement. BMD = Becker muscular dystrophy; FVC%pred = Forced Vital Capacity % predicted; LV GLS = left ventricular global longitudinal strain; NSAA = North Star Ambulatory Assessment.

The mean PUL1.2 score at first visit was 69.5 (±11 SD). Twenty-five participants (69%) achieved a maximum PUL score of 74 points throughout the follow-up period, of whom 1 was nonambulant. Out of the 11 participants with submaximal PUL1.2 scores, 7 were nonambulant at baseline and 3 lost ambulation during follow-up. Individual trajectories for the PUL grouped by NSAA score obtained at first visit are shown in Figure 3, highlighting the apparent ceiling effect of the PUL and that submaximal scoring predominantly occurs in the subgroup with a baseline NSAA score below 10.

The mean FVC% predicted at first visit was 88% (±23 SD; range 19–118; n = 34). At the first visit, only 7 participants had a reduced pulmonary function (FVC%pred <80%): 6 of whom were nonambulant and the other participant lost ambulation during follow-up. Only 2 participants were using mechanical home ventilation at night and 1 additional participant met the cutoff for a referral to one of the centers for home ventilations according to Dutch guidelines (FVC%pred <50%). We observed both nonambulant participants with normal pulmonary function as well as participants with in the NSAA 33–34 subgroup with FVC%pred below 80%. There was no clear linear decline during follow-up. Individual trajectories for the pulmonary function grouped by NSAA score at first visit are shown in Figure 3.

Echocardiography was performed in 33 participants at the first visit (23), within 6 months of the first visit (7) or at the third visit (3). A follow-up echocardiography was performed in 25 participants after 2.1 years ( ± 0.5 SD). At the first echocardiography, the mean LVEF was 46.9% (±11.3 SD; range 25–70) and the mean LV GLS was −14.8% (±3.8 SD; range −21 to −6). Individual trajectories for LV GLS grouped by NSAA score obtained at corresponding visits are shown in Figure 3. The proportion of participants with reduced LV GLS at first ultrasound (>−18%) was not different between NSAA performance subgroups (χ^2^ *p* = 0.39).

## Discussion

With the arrival of clinical trials, there is a need for outcome markers responsive to change in patients with BMD. In this natural history study, we describe the disease course in a cohort of 36 patients with BMD over a period of 3 years. We aimed to explore the sensitivity of functional outcome measures to improve trial readiness. Furthermore, we explored if sensitivity could be increased by selecting patient groups based on a specific genotype or phenotype. Our cohort comprised an extensive part of the adult disease spectrum, including nonambulant participants who had lost ambulation up to a decade before inclusion, participants who lost ambulation throughout the course of this study, and participants with minimal symptoms who obtained maximum scores on the NSAA throughout the study. In this diverse group, we found that RFFv was the only outcome measure sensitive to change at 3-year follow-up based on the SRM. Selecting an ambulant subgroup with NSAA scores between 10 and 32 at baseline resulted in a high SRM for the RFFv at 1-year follow-up and for the NSAA and 4SCv at 2-year follow-up.

Few natural history studies in patients with BMD have reported sensitivity of functional outcome measures. A Belgian study evaluated several clinical outcome measures along with MRI and patient-reported outcome measures with 9 and 18 months of follow-up in 21 ambulant, symptomatic adults with BMD.^26^ They reported a significant decline of the NSAA, 10MWRv, 6MWT, and 4SCv, all with a moderate sensitivity to change (SRM 0.4–0.78), and no significant decline of the RFFv. Especially the decline in NSAA was consistent with our findings in the functional subgroup, that is, 1.3 ± 2.0 and 2.5 ± 3.2 at 9 and 18 months vs 2.14 ± 3.08 and 2.77 ± 3.37 in 1 and 2 years in our data. Our functional subgroup seems comparable with this cohort, since they excluded nonambulant and asymptomatic participants. In our functional subgroup, NSAA showed a moderate sensitivity to change at 1 year, comparable with the Belgian study, increasing to high sensitivity to change at 2- and 3- year follow-up. By contrast, we found that the 6MWT did not reach the threshold for high sensitivity to change, whereas the RFFv already had a high sensitivity to change at just 1-year follow-up. An Italian study in 69 pediatric and adult patients with BMD with 1-year follow-up only reported a significant decline of NSAA (0.9 ± 1.6) and not of 6MWT, 10MWRv, RFFv, or 4SCv.^16^ This decline in NSAA score is comparable with the decline we observed in our whole cohort of 1.36 ± 2.45 at 1-year follow-up. A third natural history study including 83 pediatric and adult participants across multiple countries reported a decline with age for the NSAA in adult BMD patients but observed a ceiling effect of this test in participants younger than 18 years.^27^ In this study, we only included adults. Therefore, the development of sensitive outcome measures in the pediatric BMD population and high performing adults remains needed and may well include biomarkers such as quantitative MRI that is able to detect changes in fact fraction before loss of muscle function.^28-30^

Taken together, although RFFv proved the only responsive outcome measure at 1-year follow-up in ambulant adult participants with BMD, the ability to perform RFF was lost relatively early and data from different cohorts were not consistent. By contrast, the NSAA showed a high responsiveness in ambulant participants with a score at baseline between 10 and 32 at 2 and 3 years of follow-up, and the decline of 2.14 point in 1 year was similar to that reported in other studies. Moreover, NSAA could be tested in all ambulant participants. Both the NSAA and RFF, which is a task within the NSAA, were shown to be reliable and valid in measures of ambulatory function in DMD.^31^ The minimal clinically important difference (MCID) of these tests was estimated between 2.3 and 2.9 points for the NSAA^32^ and –0.212 m/sec for the RFFv^33^ in DMD boys. The change observed in our functional subgroup approximates the MCID for NSAA after 2 years, while the observed change in RFFv is lower. Although the NSAA has already been accepted as a valid primary end point in DMD and BMD trials by regulators, the MCID has not been defined in the BMD population. Defining MCID for BMD would require a larger natural history cohort incorporating patient perspective in addition to clinical tests and could aid regulatory approval of new therapies. The North Star Assessment for Limb-girdle type muscular dystrophies is a modified version of the NSAA aiming to be more sensitive to subtle progression by including extra items, for example, squatting and hip flexion in supine position, but also items reflecting axial functions such as rolling and reaching forward.^34^ This tool may further improve the responsiveness to change currently observed with the NSAA and is thereby a promising functional outcome measure for BMD to be tested in further natural history studies. Eventually, which outcome measure is preferable in clinical trials dependents on the mechanism of action of the therapeutic agent. Since current therapeutic developments are predominantly aimed at preserving muscle function and slowing decline, in this article, we analyzed decline on functional scales. In regard to trials investigating therapeutic agents aimed at improvement of function, other outcome measures may be more suitable.

In the light of exon skipping trials for DMD, interest has grown in characterizing genotype-phenotype relations in BMD. Deletion of exons 45 to 47^35^ and deletions starting at exon 45^16,17^ have been described as a typical BMD phenotype with a consistent decline with age. In our data, we did not establish a clear distinction in performance of the functional tests for the del 45–47 subgroup. A plausible explanation is that genotypes associated with a milder disease course, for example, deletion of single exon 48 and deletion ending on exon 51, are not represented in our cohort and therefor a homogenous control group for comparison is lacking. Moreover, we did not establish a more severe phenotype in the subgroup with a variant likely to affect the nNOS binding site. This might be explained by the fact that nNOS can also bind to the C-terminus of dystrophin protein through the α1-Syntrophin and thereby lead to production of NO despite a dysfunctional nNOS binding site.^36^

The PUL has been shown to be a sensitive tool to measure loss of arm function in patients with DMD and to have a good test-retest reliability in both BMD and DMD.^37,38^ However, our data showed a clear ceiling effect of the PUL1.2, with ambulant BMD participants obtaining maximum PUL scores throughout follow-up, in line with the literature.^27^ Although the nonambulant population in our study was small, we did observe a linear decline in this subgroup. Therefore, PUL1.2 could be useful when applying an entry criterium for clinical trials in the nonambulant population similar to what we show for the NSAA score in ambulant participants. Regarding pulmonary function, the FVC%pred did not show a linear decline during 3-year follow-up in most participants, complicating the use of this parameter in clinical trials. Pulmonary function was also preserved until a late stage of the disease, similar to what is reported in the literature.^10^ We previously showed that cardiac function was independent of skeletal muscle function measured with the NSAA in a cross-sectional analysis.^39^ In this study, we found that longitudinal deterioration of cardiac function and skeletal muscle function were also independent.

Limitations of the study are the relatively low SS and the fact that even with 3-year follow-up, the decline in the functional assessments could not be linked to disease milestones. Both are inherent features of slowly progressive rare neuromuscular conditions and could be resolved by collecting standardized real-world data from regular follow-up in the outpatient clinics over a substantial longer period. Specifically challenging for BMD in this regard is that more patients are followed only by cardiologists and outside expertise centers compared with DMD. We therefore initiated a nationwide disease registry for all patients with dystrophinopathies in the Netherlands that allows remote follow-up via online questionnaires.^22^ This registry also allowed us to demonstrate that participants were comparable with none participants in the current protocol regarding the genetic variants and age at LoA.^25^ The burden of the protocol was one of the main reasons to decline participation and also led to some drop-outs and missed visits in this study. We attempted to minimize the burden by scheduling all the tests on a single day and offering comfort breaks. We also decided not to repeat echocardiography if there was a measurement available obtained during routine clinical care. This meant echocardiography did not always coincide with the functional tests and was not available for all participants at baseline. Recruitment in clinical studies on BMD may also be challenging due to the specific neuropsychiatric profile that resembles that of DMD, and in which both autism spectrum disorders, anxiety, and depression can be observed.^40^

In summary, in our BMD population, RFFv at 3-year follow-up was the only functional test with a high responsiveness to change. We explored the additional value of selecting on genotype and phenotype. Although these results should be interpreted with care because of the relatively small SS, selecting on phenotype resulted in higher SRMs, whereas selecting on genotype does not seem to improve the responsiveness to change. In our functional subgroup with NSAA between 10 and 32 at baseline, RFFv showed a good responsiveness at 1-year follow-up in addition to 4SCv and NSAA at 2-year follow-up. The use of the NSAA is preferable because the NSAA can be conducted in all ambulant participants and the reported decline is consistent throughout the literature. In the design of clinical trials, we would therefore recommend to both select on NSAA scores at baseline and to use the NSAA to monitor progression. There remains an urgency to develop sensitive outcome parameters for the nonambulant, pediatric, and high performing BMD population.

## Glossary

4SCv: 4-stair climb velocity
4SDv: 4-stair descend velocity
6MWT: 6-minute walk test
10MWRTv: 10 m walk run test velocity
BMD: Becker muscular dystrophy
BMI: body mass index
DDD: Dutch Dystrophinopathy Database
DMD: Duchenne muscular dystrophy
FVC%pred: Forced Vital Capacity % predicted
LoA: loss of ambulation
LUMC: Leiden University Medical Center
LVEF: left ventricular ejection fraction
LV GLS: left ventricular global longitudinal strain
MCID: minimal clinically important difference
nNOS: neuronal nitric oxide synthesis
NO: nitric oxide
NSAA: North Star Ambulatory Assessment
PUL: Performance of Upper Limb
RFFv: rise from floor velocity
SRM: standardized response mean
SS: sample size
